# Periodontal Disease and Incident Chronic Bronchitis: A Nationwide Population-Based Cohort Study

**DOI:** 10.3390/biomedicines14091999

**Published:** 2026-09-05

**Authors:** Seon-Ju Sim, Ja-Young Moon, Hye-Sun Shin, Min-Hee Hong, Sun-Mi Kim

**Affiliations:** 1 Department of Dental Hygiene, Baekseok University, Cheonan 31065, Republic of Korea; vision1991@bu.ac.kr (S.-J.S.); mini8265@bu.ac.kr (M.-H.H.); 2 College of Medicine, Yonsei University, Seoul 03722, Republic of Korea; mjy0113@daum.net; 3 Department of Dental Hygiene, Dongnam Health University, Suwon 16328, Republic of Korea; prevoralhealth@gmail.com; 4 Department of Dental Hygiene, Wonkwang University, Iksan 54538, Republic of Korea

**Keywords:** chronic bronchitis, gingivitis, national health insurance, periodontal treatment, periodontitis, retrospective cohort study

## Abstract

**Background/Objectives:** Periodontal inflammation has been implicated in respiratory disease, but longitudinal evidence regarding chronic bronchitis (CB) remains limited. This study examined the association between claims-based periodontal status and newly recorded CB in a nationally representative Korean cohort. **Methods:** This retrospective cohort study used Korean National Health Insurance Service–National Sample Cohort data from 2002 to 2015. Periodontal status was assessed during 2002–2004, and 189,274 adults were followed during 2005–2015. Participants were classified as No PD, Gingivitis (K05.0–K05.1), or Periodontitis-related (K05.2–K05.6). Periodontal treatment was defined as recorded scaling or root planing. Cox models estimated adjusted hazard ratios (aHRs) and 95% confidence intervals (CIs). **Results:** During follow-up, 52,955 participants (28.0%) had newly recorded CB. Periodontitis-related diagnoses were associated with a modestly higher CB risk than No PD (aHR = 1.05, 95% CI = 1.02–1.08), whereas Gingivitis was not (aHR = 1.01, 95% CI = 0.99–1.03). The association was observed among adults aged 40–59 years (aHR = 1.06, 95% CI = 1.02–1.11) and those without recorded periodontal treatment (aHR = 1.08, 95% CI = 1.03–1.12). No significant association was observed among treated participants (aHR = 0.86, 95% CI = 0.74–1.01). **Conclusions:** Periodontitis-related diagnoses were associated with a small increase in newly recorded CB risk, whereas Gingivitis was not. Treatment-stratified findings were exploratory and should not be interpreted as evidence that periodontal treatment prevents CB.

## 1. Introduction

Chronic bronchitis (CB) is commonly defined as a productive cough occurring for at least 3 months per year over two consecutive years [1]. It reflects long-standing airway inflammation and is closely linked to exposure to tobacco smoke and other inhaled irritants. CB represents a clinically meaningful symptom-based phenotype in chronic obstructive pulmonary disease (COPD), with prevalence estimates among COPD patients varying across populations and diagnostic criteria [2].

The clinical relevance of CB lies in its association with heavier symptom burden, poorer quality of life, and more frequent respiratory exacerbations than those observed in individuals without this phenotype [3]. In population-based settings, CB prevalence has been reported within a broad range, and established risk factors include older age, low educational attainment, smoking, occupational fumes, household and ambient air pollution, asthma or lung malignancy, and familial susceptibility to chronic respiratory disease [2].

Periodontal disease (PD) encompasses inflammatory conditions affecting the gingiva and tooth-supporting tissues, including gingivitis and periodontitis. Gingivitis is limited to inflammation of the gingiva, whereas periodontitis involves progressive destruction of the periodontal supporting tissues through interactions between dysbiotic subgingival microbiota and a susceptible host response [4]. Because of its high prevalence and cumulative impact on adult oral health, periodontitis is a major public health concern worldwide [5]. In South Korea, the healthcare burden of PD has continued to grow; in 2021, more than 17 million outpatient visits were attributed to PD, making it the leading cause of outpatient dental or medical visits in national statistics [6]. Epidemiologic evidence also links PD with systemic conditions including cardiovascular conditions, diabetes mellitus, obesity, hepatic disease and respiratory disorders [7,8,9,10,11].

Previous research on periodontal and respiratory health has included cross-sectional and case–control studies, prospective cohort studies, clinical trials, and systematic reviews [11,12,13,14,15,16,17,18,19]. These studies have generally reported positive associations between periodontal disease and respiratory outcomes, although the magnitude of the associations has varied across study designs and outcome definitions. Qian et al. [20] reported that severe periodontitis was associated with respiratory disease mortality (adjusted HR, 2.72; 95% CI, 1.04–7.11). A systematic review and meta-analysis by Gomes-Filho et al. [11] reported a pooled adjusted OR of 1.78 (95% CI, 1.04–3.05) for the association between periodontitis and COPD.

Evidence specifically addressing the onset of CB among individuals with periodontal disease remains limited, particularly in longitudinal population-based settings. Alterations in oral microbial and inflammatory conditions may affect the lower respiratory environment, providing a plausible basis for investigating this relationship [21]. The present study therefore examined whether baseline periodontal status, classified as no recorded periodontal disease, gingivitis-related diagnoses, or periodontitis-related diagnoses, was associated with subsequent CB and whether the association varied according to demographic characteristics and periodontal treatment.

## 2. Materials and Methods

### 2.1. Study Database and Ethical Considerations

Data for this retrospective population-based cohort analysis were obtained from the Korean National Health Insurance Service–National Sample Cohort (NHIS-NSC). The database contains longitudinal insurance eligibility, healthcare utilization, diagnostic, treatment, and health examination information for approximately one million individuals, corresponding to about 2.2% of the Korean population. Records collected between 2002 and 2015 were used in the present analysis [22]. The study protocol was reviewed by the Institutional Review Board of Baekseok University and was determined to be exempt from ethical review because the study used anonymized secondary data and the investigators had no access to personally identifiable information (exemption date: 29 January 2020; BUIRB-202001-HR-025). Informed consent was not required because the investigators had no access to personally identifiable information.

### 2.2. Cohort Construction and Follow-Up

**Figure 1:**
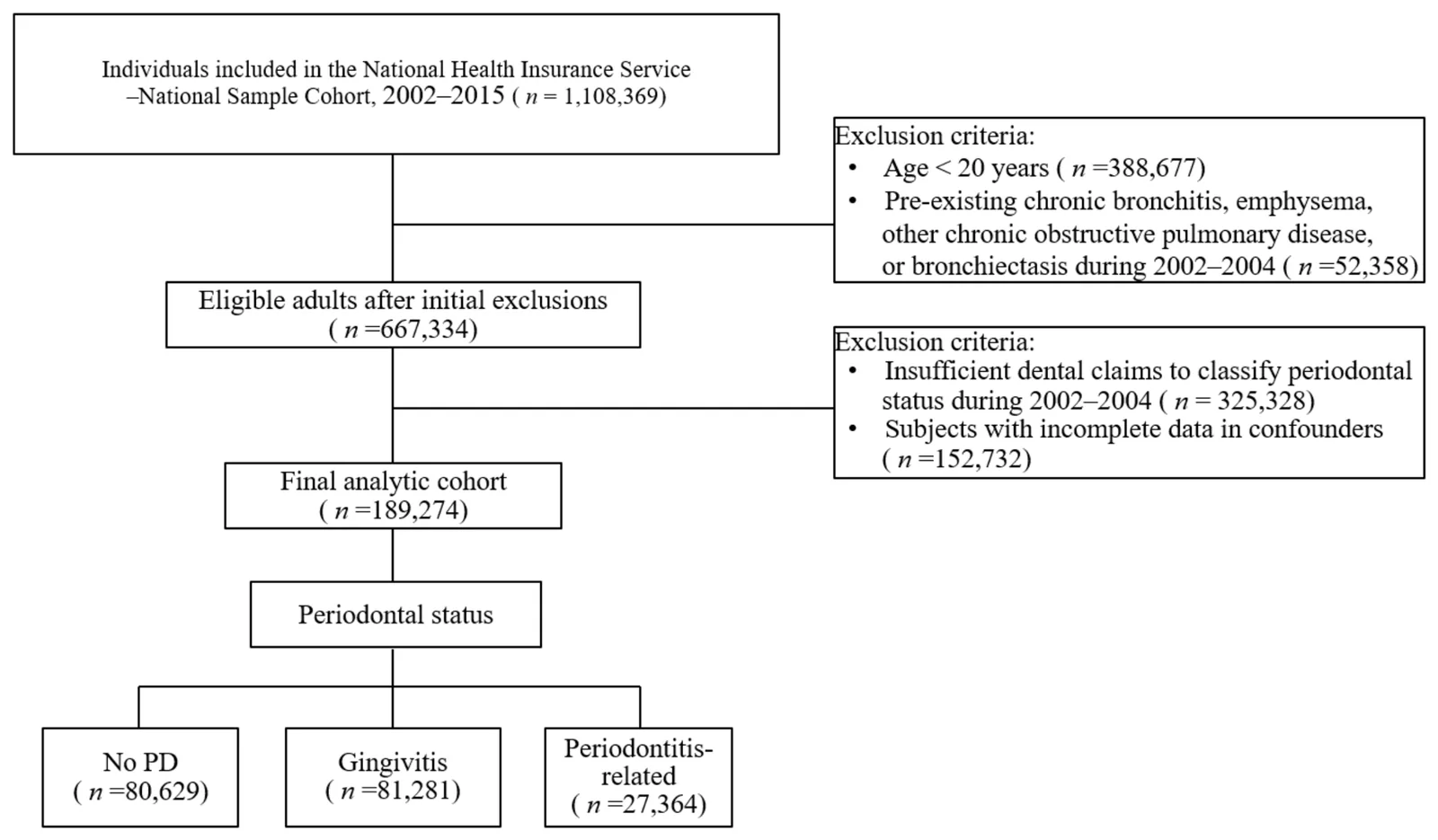
Flow diagram of study participant selection. NHIS-NSC, National Health Insurance Service–National Sample Cohort; No PD, no recorded periodontal diagnosis.

The source database included 1,108,369 individuals. Eligibility was restricted to adults aged 20 years or older. Accordingly, 388,677 individuals younger than 20 years were excluded. To identify newly diagnosed cases of chronic bronchitis, we additionally excluded 52,358 participants with a recorded diagnosis of chronic bronchitis (ICD-10 codes J41–J42), emphysema (J43), other chronic obstructive pulmonary disease (J44), or bronchiectasis (J47) during the baseline period from 2002 to 2004. Periodontal status was assessed during the baseline period from 2002 to 2004. Individuals whose records during this interval were insufficient to determine periodontal status were excluded (*n* = 325,328), as were those with incomplete information on the covariates included in the multivariable analyses (*n* = 152,732). The resulting analytic cohort consisted of 189,274 adults (Figure 1). Follow-up for incident CB began on 1 January 2005 and continued through 31 December 2015, providing up to 11 years of follow-up.

### 2.3. Periodontal Status and Treatment

Baseline periodontal status was determined using dental claims recorded by qualified dentists. Gingival and periodontal conditions were identified using International Classification of Diseases, 10th Revision (ICD-10) codes: K05.0 for acute gingivitis, K05.1 for chronic gingivitis, K05.2 for acute periodontitis, K05.3 for chronic periodontitis, K05.4 for periodontosis, K05.5 for other periodontal diseases, and K05.6 for unspecified periodontal disease. For analysis, participants were operationally classified into three groups based on periodontal diagnoses recorded during 2002–2004: no periodontal disease (No PD), defined as no recorded periodontal diagnosis; gingivitis-related diagnoses (Gingivitis), defined by K05.0–K05.1; and periodontitis-related diagnoses (Periodontitis-related), defined by K05.2–K05.6. These claims-based categories were used for analytic purposes and should not be interpreted as clinically determined stages or grades of periodontitis. In addition, No PD indicates the absence of a recorded periodontal diagnosis rather than the confirmed absence of periodontal disease. Periodontal treatment was defined as recorded scaling (U2232) or root planing (U2240) during the baseline period.

### 2.4. Chronic Bronchitis Outcome

The study outcome was the first diagnosis of chronic bronchitis recorded during follow-up. Chronic bronchitis was identified using ICD-10 codes J41 and J42. Participants with a corresponding diagnosis before follow-up were excluded to ensure that the analyses reflected newly recorded cases.

### 2.5. Covariates

Variables considered as potential confounders were selected before the analyses. Demographic and socioeconomic factors included age, sex, and household income. Household income was divided into quintiles according to the insurance premiums assigned to each household.

Health-related behaviors included smoking and alcohol consumption. Smoking was classified as yes or no. Alcohol consumption was categorized as almost never, four or fewer times per week, or almost every day, based on responses to the national health examination questionnaire. The health conditions included in the adjusted models were diabetes mellitus, hypertension, obesity, and hypercholesterolemia. Diabetes mellitus was identified using ICD-10 codes E10–E14, and hypertension using ICD-10 codes I10 and I15. Obesity was defined as a body mass index of at least 25.0 kg/m^2^ [23]. Hypercholesterolemia was defined as a total serum cholesterol level of ≥240 mg/dL. Smoking status, alcohol consumption, body mass index, and serum cholesterol levels were obtained from the most recent health examination conducted during the baseline period.

### 2.6. Statistical Analysis

Baseline characteristics according to periodontal status were summarized as frequencies and percentages and compared using the chi-square test. CB incidence rates were calculated according to periodontal status and expressed per 1000 person-years. Person-years were calculated from the start of follow-up to the date of incident chronic bronchitis or the end of the study period, whichever occurred first. Kaplan–Meier curves and log-rank tests were used to compare CB-free survival across periodontal status groups. Cox proportional hazards models estimated the association between periodontal status and incident CB over the 11-year follow-up period. Hazard ratios (HRs), adjusted hazard ratios (aHRs), and 95% confidence intervals (CIs) were estimated sequentially. Model 1 was unadjusted. Model 2 adjusted for age, sex, and household income. Model 3 additionally adjusted for smoking status and alcohol consumption. Model 4 further adjusted for diabetes mellitus, hypertension, obesity, and hypercholesterolemia. Subgroup analyses were performed by age group, sex, and periodontal treatment status. All analyses were performed using IBM SPSS Statistics for Windows, version 28.0 (IBM Corp., Armonk, NY, USA). All statistical tests were two-sided, and a *p*-value < 0.05 was considered statistically significant. 

## 3. Results

### 3.1. Participant Characteristics and Occurrence of Chronic Bronchitis

The final analysis included 189,274 adults. At baseline, 42.6% of participants were classified as No PD, 42.9% as Gingivitis, and 14.5% as Periodontitis-related (Table 1). Baseline characteristics differed significantly across the three periodontal status groups (all *p* < 0.001). Compared with participants with No PD, the Gingivitis and Periodontitis-related groups included greater proportions of older adults, men, smokers, and participants who consumed alcohol more frequently. Diabetes mellitus, hypertension, obesity, and hypercholesterolemia also differed significantly across the three groups. During follow-up, 52,955 participants received a new diagnosis of CB, representing 28.0% of the cohort. The proportion developing CB was 26.6% in the No PD group, 28.3% in the Gingivitis group, and 31.1% in the Periodontitis-related group (*p* < 0.001).

### 3.2. Crude Association

**Figure 2:**
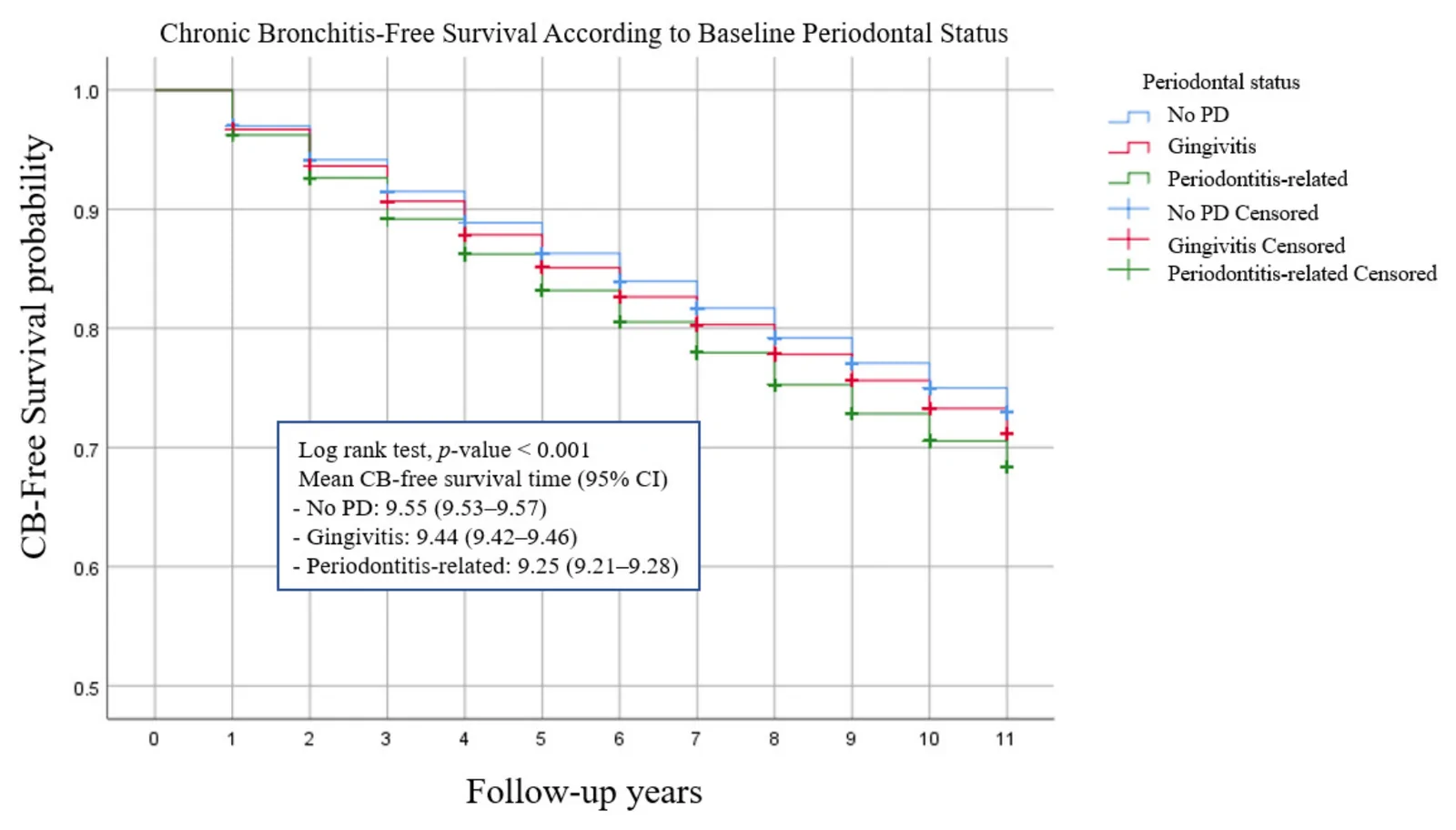
Kaplan–Meier curves for chronic bronchitis-free survival according to baseline periodontal status. Differences among groups were evaluated using the log-rank test (*p* < 0.001). Tick marks indicate censored observations. CB, chronic bronchitis; No PD, no recorded periodontal diagnosis.

In the unadjusted Cox model, Periodontitis-related diagnoses were associated with a higher risk of incident CB compared with No PD (crude HR = 1.21, 95% CI = 1.18–1.24). Gingivitis was also associated with a modestly higher crude risk (crude HR = 1.08, 95% CI = 1.06–1.10). Kaplan–Meier analysis showed a higher CB-free survival probability in the No PD group than in the Gingivitis and Periodontitis-related groups (Figure 2). The estimated mean CB-free survival time was 9.55 years (95% CI, 9.53–9.57) in the No PD group, 9.44 years (95% CI, 9.42–9.46) in the Gingivitis group, and 9.25 years (95% CI, 9.21–9.28) in the Periodontitis-related group (log-rank *p* < 0.001). The cumulative incidence of CB was 26.6%, 28.3%, and 31.1% in the No PD, Gingivitis, and Periodontitis-related groups, respectively.

### 3.3. Adjusted Association

After full adjustment in Model 4, Periodontitis-related diagnoses were significantly associated with incident CB (aHR = 1.05, 95% CI = 1.02–1.08), whereas Gingivitis was not significantly associated with CB (aHR = 1.01, 95% CI = 0.99–1.03) (Table 2). In age-stratified analyses, the association with Periodontitis-related diagnoses was most apparent among participants aged 40–59 years (aHR = 1.06, 95% CI = 1.02–1.11). Sex-stratified estimates for Periodontitis-related diagnoses were similar in men (aHR = 1.05, 95% CI = 1.01–1.10) and women (aHR = 1.05, 95% CI = 1.01–1.10) (Table 3).

### 3.4. Findings According to Periodontal Treatment

Table 4 presents the subgroup analyses according to recorded periodontal treatment status. Among participants without recorded periodontal treatment, periodontitis-related diagnoses were associated with a higher risk of incident CB after full adjustment (aHR = 1.08, 95% CI = 1.03–1.12). Among participants with recorded periodontal treatment, the association between periodontitis-related diagnoses and CB was not statistically significant (aHR = 0.86, 95% CI = 0.74–1.01).

**Table 2:** Incidence Rates and Hazard Ratios for Chronic Bronchitis According to Periodontal Status.

				Model 1	Model 2	Model 3	Model 4
Independent Variable	Participants, *n*	CB Events, *n* (%)	Incidence Rate per 1000 Person-Years	cHR (95% CI)	aHR (95% CI)	aHR (95% CI)	aHR (95% CI)
Periodontal status							
No PD	80,629	21,423 (26.6)	28.37	ref	ref	ref	ref
Gingivitis	81,281	23,019 (28.3)	30.61	** 1.08 (1.06–1.10) **	1.01 (1.00–1.03)	1.01 (1.00–1.03)	1.01 (0.99–1.03)
Periodontitis- related	27,364	8513 (31.1)	34.29	** 1.21 (1.18–1.24) **	** 1.06 (1.04–1.09) **	** 1.06 (1.04–1.09) **	** 1.05 (1.02–1.08) **

Model 1: Unadjusted. Model 2: age group, sex, income. Model 3: age group, sex, income, smoking status, alcohol consumption. Model 4: age group, sex, income, smoking status, alcohol consumption, diabetes mellitus, hypertension, obesity, hypercholesterolemia. Crude hazard ratio, cHR; adjusted hazard ratio, aHR; 95% confidence interval, 95% CI. Bold denotes statistical significance at *p* < 0.05. No PD, no recorded periodontal diagnosis; Gingivitis, gingivitis-related diagnoses (K05.0–K05.1); Periodontitis-related, periodontitis-related diagnoses (K05.2–K05.6).

**Table 3:** Association of periodontal status with the incidence of chronic bronchitis by age and sex.

			Model 1	Model 2	Model 3	Model 4
Independent Variable	Participants, *n*	CB Events, *n* (%)	cHR (95% CI)	aHR (95% CI)	aHR (95% CI)	aHR (95% CI)
Sex						
Male						
No PD	39,427	9467 (24.0)	ref	ref	ref	ref
Gingivitis	42,200	10,965 (26.0)	** 1.10 (1.07–1.13) **	1.00 (0.97–1.03)	1.00 (0.97–1.03)	1.00 (0.97–1.03)
Periodontitis- related	15,329	4551 (29.7)	** 1.28 (1.23–1.32) **	** 1.05 (1.02–1.09) **	** 1.05 (1.02–1.09) **	** 1.05 (1.01–1.10) **
Female						
No PD	41,202	11,956 (29.0)	ref	ref	ref	ref
Gingivitis	39,081	12,054 (30.8)	** 1.08 (1.05–1.10) **	** 1.03 (1.00–1.05) **	1.03 (1.00–1.05)	1.02 (0.99–1.05)
Periodontitis- related	12,035	3962 (32.9)	** 1.17 (1.13–1.21) **	** 1.06 (1.02–1.10) **	** 1.06 (1.02–1.10) **	** 1.05 (1.01–1.10) **
Age group						
20–39						
No PD	37,385	7308 (19.5)	ref	ref	ref	ref
Gingivitis	29,587	5870 (19.8)	1.02 (0.98–1.05)	1.02 (0.98–1.05)	1.02 (0.98–1.05)	1.02 (0.98–1.06)
Periodontitis- related	6510	1335 (20.5)	1.05 (0.99–1.11)	1.05 (0.99–1.11)	1.05 (0.99–1.11)	1.05 (0.98–1.12)
40–59						
No PD	33,092	9722 (29.4)	ref	ref	ref	ref
Gingivitis	38,346	11,338 (29.6)	1.01 (0.98–1.04)	1.02 (1.00–1.05)	1.02 (1.00–1.05)	1.02 (0.99–1.05)
Periodontitis- related	15,110	4610 (30.5)	** 1.05 (1.01–1.09) **	** 1.08 (1.04–1.12) **	** 1.08 (1.04–1.12) **	** 1.06 (1.02–1.11) **
≥60 y						
No PD	10,152	4393 (43.3)	ref	ref	ref	ref
Gingivitis	13,348	5811 (43.5)	1.00 (0.96–1.04)	1.00 (0.96–1.04)	1.00 (0.96–1.04)	1.00 (0.96–1.05)
Periodontitis- related	5744	2568 (44.7)	1.04 (0.99–1.09)	1.04 (0.99–1.09)	1.04 (0.99–1.09)	1.04 (0.98–1.10)

Model 1: Unadjusted. Model 2: age group, sex, income. Model 3: age group, sex, income, smoking status, alcohol consumption. Model 4: age group, sex, income, smoking status, alcohol consumption, diabetes mellitus, hypertension, obesity, hypercholesterolemia. For sex-stratified analyses, sex was omitted from the corresponding adjustment models; for age-stratified analyses, age group was omitted. Crude hazard ratio, cHR; adjusted hazard ratio, aHR; 95% confidence interval, 95% CI. Bold denotes statistical significance at *p* < 0.05. No PD, no recorded periodontal diagnosis; Gingivitis, gingivitis-related diagnoses (K05.0–K05.1); Periodontitis-related, periodontitis-related diagnoses (K05.2–K05.6).

**Table 4:** Association of periodontal status with the incidence of chronic bronchitis by periodontal treatment in the Cox proportional hazards models.

			Model 1	Model 2	Model 3	Model 4
Independent Variable	Participants, *n*	CB Events, *n* (%)	cHR (95% CI)	aHR (95% CI)	aHR (95% CI)	aHR (95% CI)
Without periodontal treatment						
No PD	80,133	21,262 (26.5)	ref	ref	ref	ref
Gingivitis	47,456	13,432 (28.3)	**1.08 (1.06–1.10)**	**1.02 (1.00–1.04)**	**1.02 (1.00–1.04)**	1.01 (0.99–1.03)
Periodontitis-related	8070	2578 (31.9)	**1.26 (1.21–1.31)**	**1.10 (1.06–1.15)**	**1.10 (1.05–1.15)**	**1.08 (1.03–1.12)**
With periodontal treatment						
No PD	496	161 (32.5)	ref	ref	ref	ref
Gingivitis	33,825	9587 (28.3)	0.87 (0.74–1.01)	0.84 (0.72–0.98)	0.84 (0.72–0.98)	0.84 (0.72–0.98)
Periodontitis- related	19,294	5935 (30.8)	0.96 (0.82–1.12)	0.87 (0.74–1.02)	0.87 (0.74–1.02)	0.86 (0.74–1.01)

Model 1: Unadjusted. Model 2: age group, sex, income. Model 3: age group, sex, income, smoking status, alcohol consumption. Model 4: age group, sex, income, smoking status, alcohol consumption, diabetes mellitus, hypertension, obesity, hypercholesterolemia. Crude hazard ratio, cHR; adjusted hazard ratio, aHR; 95% confidence interval, 95% CI. Bold denotes statistical significance at *p* < 0.05. No PD, no recorded periodontal diagnosis; Gingivitis, gingivitis-related diagnoses (K05.0–K05.1); Periodontitis-related, periodontitis-related diagnoses (K05.2–K05.6).

## 4. Discussion

In this nationwide cohort, periodontitis-related diagnoses were associated with a small but statistically significant increase in the risk of newly recorded CB, whereas gingivitis was not associated with CB after adjustment. The association was also observed among adults aged 40–59 years and participants without recorded periodontal treatment, whereas no significant association was observed among those with recorded periodontal treatment. Given the modest effect size and observational design, these subgroup findings should be considered exploratory and should not be interpreted as evidence of causality or a preventive effect of periodontal treatment.

The baseline profiles across periodontal-status groups were consistent with known epidemiologic patterns. Older age, male sex, smoking, frequent alcohol consumption, and cardiometabolic conditions were more common across the No PD, gingivitis, and periodontitis-related groups. The clustering of periodontal disease with socioeconomic and cardiometabolic factors highlights the importance of controlling for shared determinants when evaluating periodontal-respiratory associations [9,10,24,25].

Although the direction of the association observed in our study was consistent with previous reports, the magnitude was substantially smaller than that reported in several earlier studies. Qian et al. [20], for example, reported an adjusted HR of 2.72 for respiratory disease mortality among individuals with severe periodontitis, whereas Gomes-Filho et al. [11] reported a pooled adjusted OR of 1.78 for the association between periodontitis and COPD. Shen et al. [26] reported lower rates of adverse respiratory events among COPD patients with periodontal disease who received periodontal treatment compared with propensity-matched COPD patients without periodontal disease, including hospitalization (adjusted IRR, 0.74; 95% CI, 0.69–0.80) and all-cause mortality (adjusted RR, 0.57; 95% CI, 0.52–0.62). In contrast, the present study assessed incident chronic bronchitis according to baseline periodontal status in a general population cohort. These differences in study population, exposure definition, and respiratory outcome may partly explain the differences in effect estimates across studies. Chronic bronchitis may represent a broader and less specific clinical phenotype than endpoints such as COPD-related adverse events or respiratory mortality. Differences in periodontal disease ascertainment, follow-up duration, and covariate adjustment may also have contributed to variation in effect estimates across studies.

Patients with the CB phenotype generally experience a greater clinical burden than those with COPD without CB, including more frequent exacerbations and less favorable outcomes [27]. Heightened airway and systemic inflammatory activity may contribute to this increased burden and provide clinical context for investigating periodontal inflammation as a potential correlate of incident CB.

The association was most evident among adults aged 40–59 years. In older adults, accumulated smoking exposure, cardiometabolic disease, and other respiratory risk factors may have reduced the relative contribution of periodontal status. However, because the difference between age groups was small, this finding should not be interpreted as definitive evidence of effect modification. Further longitudinal research incorporating inflammatory biomarkers and more detailed measures of lifetime exposure is required to clarify this pattern.

Despite known sex-related differences in periodontal and respiratory health, the adjusted association between periodontitis-related diagnoses and CB was nearly identical in men and women. Sex-related differences in adiposity, hormonal factors, healthcare utilization, and systemic inflammatory responses may influence periodontal health [28]. Women with COPD may also be more likely to present with the chronic bronchitis phenotype [29]. Nevertheless, the similar estimates observed in the present study suggest that the association between periodontitis-related diagnoses and incident CB may not differ substantially by sex after adjustment for major confounders.

Several biological mechanisms may plausibly link periodontal disease with chronic airway inflammation. Chronic microaspiration can lead to overlap between oral and pulmonary microbial communities, supporting the concept of an oral–lung axis [30]. Oral dysbiosis and periodontal inflammation may also influence host defense and inflammatory responses relevant to the lower airways [30,31]. Aspiration of oral microorganisms and systemic dissemination of periodontal inflammatory mediators may contribute to sustained airway inflammation [30,31]. Protease-mediated tissue injury represents another potential mechanism shared by periodontal destruction and chronic pulmonary disease [32].

By reducing plaque accumulation, periodontal pathogen burden, and local inflammatory activity, periodontal care may limit repeated microbial aspiration and systemic inflammatory stimulation. Dental plaque has been identified as a potential reservoir for respiratory pathogens in vulnerable patients, providing indirect support for this pathway [33]. Clinical studies have also reported fewer COPD exacerbations or adverse respiratory events following periodontal treatment, although the available evidence remains limited [17,26]. The multiple-hit hypothesis suggests that chronic inflammatory stimuli outside the airway may contribute to the development or progression of severe airway disease [34]. Periodontal inflammation could represent one such additional inflammatory stimulus, although this mechanism remains hypothetical.

An important secondary finding was the difference observed according to recorded periodontal treatment. Among participants without recorded periodontal treatment, periodontitis-related diagnoses were associated with a higher risk of newly recorded CB, whereas no significant association was observed for periodontitis-related diagnoses among those with recorded periodontal treatment. In the stratified analyses, the association between periodontitis-related diagnoses and CB was statistically significant among participants without recorded periodontal treatment but not among those with recorded periodontal treatment. However, because treatment was not randomly assigned, no formal interaction test was performed, and the No PD reference group among participants with recorded periodontal treatment included only 496 participants, the treatment-stratified findings should be interpreted cautiously. The small reference group may have limited the statistical precision and stability of the estimates. Accordingly, these findings should be regarded as hypothesis-generating rather than evidence of a causal protective effect of periodontal treatment.

Participants who received periodontal care may have differed from untreated participants in health awareness, healthcare access, socioeconomic status, oral hygiene behavior, management of chronic conditions, baseline periodontal characteristics, or frequency of dental attendance. These differences may have contributed to residual confounding. Therefore, further studies are needed to determine whether periodontal treatment modifies the association between periodontal status and CB.

This study has several strengths. The analysis used a large nationwide cohort with an 11-year follow-up period and established temporal separation between periodontal assessment and ascertainment of newly recorded CB. Periodontal and respiratory diagnoses were obtained from healthcare records generated by dental and medical professionals. The sample size also permitted analyses according to periodontal status, age, sex, and treatment status. In addition, sequential adjustment models demonstrated how the association changed after accounting for major shared risk factors.

Several limitations should also be considered. First, periodontal status was identified from claims-based diagnostic codes rather than standardized clinical measurements such as probing depth, clinical attachment level, bleeding on probing, or radiographic bone loss. The claims-based categories therefore reflect recorded gingivitis and periodontitis-related diagnoses and do not provide direct information on clinical periodontal severity or periodontitis stage and grade. Differences in coding practices and patterns of dental attendance may also have resulted in exposure misclassification.

Second, CB was identified from claims-based diagnostic codes rather than symptom questionnaires, spirometry, imaging, or review of clinical records. Some individuals with chronic cough and sputum may not have sought medical care, while others may have been coded differently within the spectrum of chronic respiratory disease. The findings consequently apply to clinically recorded CB rather than all symptom-defined cases in the population.

Third, although the analyses adjusted for smoking status, the available information did not fully capture smoking duration, cumulative exposure, passive smoking, changes in smoking behavior, or occupational and environmental inhalational exposures. Residual confounding by tobacco use and air pollution is therefore likely. Information on oral hygiene practices, tooth loss, dental plaque, socioeconomic circumstances beyond insurance premium, medication use, and respiratory function was also unavailable.

Fourth, periodontal exposure and treatment were assessed during the baseline period and were not modeled as time-varying factors. Participants may have experienced progression, remission, additional treatment, or changes in oral health during follow-up. Similarly, the observational design cannot exclude surveillance bias, as individuals with more frequent healthcare contact may have had a greater probability of receiving both periodontal and respiratory diagnoses.

Fifth, ascertainment of incident CB began immediately after the 2002–2004 baseline period, without an additional lag interval. Consequently, subclinical respiratory disease already present near the end of the baseline period may not have been fully excluded, and the possibility of reverse causation cannot be ruled out.

Sixth, substantial exclusions due to insufficient dental claims or missing covariate data may have introduced selection bias, and differences between included and excluded individuals could not be fully assessed.

Finally, the study population was drawn from the Korean National Health Insurance system. Differences in disease prevalence, healthcare utilization, smoking patterns, and access to dental care may limit direct generalization to other countries or healthcare settings.

## 5. Conclusions

In this nationwide cohort, periodontitis-related diagnoses were modestly associated with an increased risk of newly recorded CB over 11 years of follow-up, whereas gingivitis-related diagnoses were not significantly associated with CB after multivariable adjustment. The association with periodontitis-related diagnoses was observed among middle-aged adults and participants without recorded periodontal treatment, whereas no significant association was observed among those with recorded periodontal treatment. However, because no formal interaction test was performed, the treatment-stratified findings should be considered exploratory and should not be interpreted as evidence that periodontal treatment modifies or prevents CB. Further studies using clinical periodontal assessments are needed to confirm these findings.

## Figures and Tables

**Table 1:** Baseline characteristics of the study participants according to claims-based periodontal status (N = 189,274).

		Periodontal Status			
Variables	Total	No PD (*n* = 80,629)	Gingivitis (*n* = 81,281)	Periodontitis- Related (*n* = 27,364)	*p*-Value ^1^
Age group					
20–39	73,482	37,385 (50.9)	29,587 (40.2)	6510 (8.9)	**<0.001**
40–59	86,548	33,092 (38.2)	38,346 (44.3)	15,110 (17.5)	
≥60 years	29,244	10,152 (34.8)	13,348 (45.6)	5744 (19.6)	
Sex					
Male	96,956	39,427 (40.7)	42,200 (43.5)	15,329 (15.8)	**<0.001**
Female	92,318	41,202 (44.6)	39,081 (42.3)	12,035 (13.1)	
Household income quintiles					
First quintile	25,622	11,470 (44.8)	10,927 (42.6)	3225 (12.6)	**<0.001**
Second quintile	29,227	13,023 (44.6)	12,610 (43.1)	3594 (12.3)	
Third quintile	40,747	17,798 (43.7)	17,543 (43.0)	5406 (13.3)	
Fourth quintile	42,812	17,930 (41.9)	18,500 (43.2)	6382 (14.9)	
Fifth quintile	50,866	20,408 (40.1)	21,701 (42.7)	8757 (17.2)	
Smoking status					
No	128,697	55,233 (42.9)	55,022 (42.8)	18,442 (14.3)	**<0.001**
Yes	60,577	25,396 (41.9)	26,259 (43.4)	8922 (14.7)	
Alcohol consumption					
Rarely/never	98,850	41,505 (42.0)	42,591 (43.1)	14,754 (14.9)	**<0.001**
≤4 times/week	84,646	37,089 (43.8)	35,920 (42.4)	11,637 (13.8)	
Almost every day	5778	2035 (35.3)	2770 (47.9)	973 (16.8)	
Diabetes mellitus					
No	166,400	72,811 (43.8)	70,984 (42.7)	22,605 (13.5)	**<0.001**
Yes	22,874	7818 (34.2)	10,297 (45.0)	4759 (20.8)	
Hypertension					
No	163,385	71,614 (43.8)	69,604 (42.6)	22,167 (13.6)	**<0.001**
Yes	25,889	9015 (34.8)	11,677 (45.1)	5197 (20.1)	
Obesity					
No	129,194	55,729 (43.1)	55,234 (42.8)	18,231 (14.1)	**<0.001**
Yes	60,080	24,900 (41.4)	26,047 (43.4)	9133 (15.2)	
Hypercholesterolemia					
No	176,157	75,193 (42.7)	75,540 (42.9)	25,424 (14.4)	**<0.001**
Yes	13,117	5436 (41.4)	5741 (43.8)	1940 (14.8)	

Data are presented as *n* (row %), unless otherwise indicated. No PD, no recorded periodontal diagnosis; Gingivitis, gingivitis-related diagnoses (K05.0–K05.1); Periodontitis-related, periodontitis-related diagnoses (K05.2–K05.6). ^1^ *p*-values were obtained from overall chi-square tests across the three periodontal status groups. Bold denotes statistical significance at *p* < 0.05.

## Data Availability

Restrictions apply to the availability of these data. The data were obtained from the Korean National Health Insurance Service and are available through the NHIS data access system upon application and approval by the NHIS. The authors are not permitted to share or redistribute the data.
